# Feasibility, Biodistribution, and Preliminary Dosimetry in Peptide-Targeted Radionuclide Therapy of Diverse Adenocarcinomas Using ^177^Lu-FAP-2286: First-in-Humans Results

**DOI:** 10.2967/jnumed.120.259192

**Published:** 2022-03

**Authors:** Richard P. Baum, Christiane Schuchardt, Aviral Singh, Maythinee Chantadisai, Franz C. Robiller, Jingjing Zhang, Dirk Mueller, Alexander Eismant, Frankis Almaguel, Dirk Zboralski, Frank Osterkamp, Aileen Hoehne, Ulrich Reineke, Christiane Smerling, Harshad R. Kulkarni

**Affiliations:** 1 Theranostics Center for Molecular Radiotherapy and Molecular Imaging, Zentralklinik Bad Berka, Bad Berka, Germany;; 2 Curanosticum Wiesbaden–Frankfurt, Center for Advanced Radiomolecular Precision Oncology, Wiesbaden, Germany;; 3 Faculty of Medicine, Chulalongkorn University, King Chulalongkorn Memorial Hospital, Bangkok, Thailand;; 4 Yong Loo Lin School of Medicine, National University of Singapore, Singapore, Singapore;; 5 Loma Linda University, Loma Linda, California; and; 6 3B Pharmaceuticals GmbH, Berlin, Germany

**Keywords:** fibroblast activation protein, ^177^Lu-FAP-2286, peptide-targeted radionuclide therapy, first-in-humans, adenocarcinoma

## Abstract

Fibroblast activation protein (FAP) is a promising target for diagnosis and therapy of numerous malignant tumors. FAP-2286 is the conjugate of a FAP-binding peptide, which can be labeled with radionuclides for theranostic applications. We present the first-in-humans results using ^177^Lu-FAP-2286 for peptide-targeted radionuclide therapy (PTRT). **Methods:** PTRT using ^177^Lu-FAP-2286 was performed on 11 patients with advanced adenocarcinomas of the pancreas, breast, rectum, or ovary after prior confirmation of uptake on ^68^Ga-FAP-2286 or ^68^Ga-FAPI-04 PET/CT. **Results:** Administration of ^177^Lu-FAP-2286 (5.8 ± 2.0 GBq; range, 2.4–9.9 GBq) was well tolerated, with no adverse symptoms or clinically detectable pharmacologic effects being noticed or reported in any of the patients. The whole-body effective dose was 0.07 ± 0.02 Gy/GBq (range, 0.04–0.1 Gy/GBq). The mean absorbed doses for kidneys and red marrow were 1.0 ± 0.6 Gy/GBq (range, 0.4–2.0 Gy/GBq) and 0.05 ± 0.02 Gy/GBq (range, 0.03–0.09 Gy/GBq), respectively. Significant uptake and long tumor retention of ^177^Lu-FAP-2286 resulted in high absorbed tumor doses, such as 3.0 ± 2.7 Gy/GBq (range, 0.5–10.6 Gy/GBq) in bone metastases. No grade 4 adverse events were observed. Grade 3 events occurred in 3 patients—1 with pancytopenia, 1 with leukocytopenia, and 1 with pain flare-up; 3 patients reported a pain response. **Conclusion:** ^177^Lu-FAP-2286 PTRT, applied in a broad spectrum of cancers, was relatively well tolerated, with acceptable side effects, and demonstrated long retention of the radiopeptide. Prospective clinical studies are warranted.

## Visual Abstract

**Figure:**
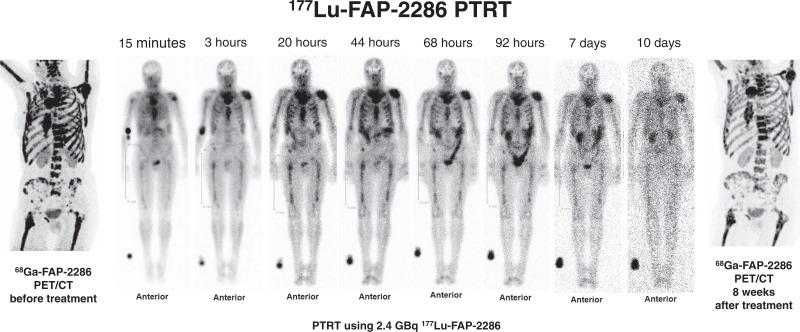

In recent years, the tumor microenvironment has gained interest as a therapeutic target in the treatment of cancer. The tumor microenvironment can comprise a substantial part of the tumor: in pancreatic ductal carcinoma, for example, it has been reported to form up to 80% of the tumor mass (*1*). Cancer-associated fibroblasts are an integral part of the tumor microenvironment and present abundantly in the stroma of tumor entities such as breast and colon cancer (*2*). A marker protein of cancer-associated fibroblasts is fibroblast activation protein (FAP), a type II transmembrane cell surface serine proteinase belonging to the dipeptidyl peptidase family (*3*). FAP, which was discovered more than 30 y ago, is overexpressed on the cancer-associated fibroblasts of over 90% of epithelial tumors such as breast, colorectal, lung, ovarian, and pancreatic adenocarcinomas. Since FAP expression in normal tissue is limited, it has been identified as a pan-tumor target for the potential treatment of cancer (*4*). In cancer indications of mesenchymal origin, notably sarcoma and mesothelioma, FAP is expressed on the tumor cells in addition to cancer-associated fibroblasts (*5*,*6*). FAP expression is also present in chronic inflammatory diseases such as rheumatoid arthritis and osteoarthritis, as well as during cardiac remodeling after myocardial infarction (*7*,*8*).

The first approach toward using FAP as a target in cancer treatment applied the monoclonal antibody sibrotuzumab and was tested in both an unconjugated and a ^131^I-conjugated format for colorectal cancer (*9*,*10*). Other approaches targeting FAP, including bispecific antibodies or antibody fragment constructs, chimeric antigen receptor T cells, and antibody–drug conjugates, are currently being pursued, most of which are in preclinical development or phase 1 clinical trials (*6*,*11*). Small-molecule FAP inhibitors (FAPIs) have also been discovered (*12*) and conjugated with radioactivity to yield excellent imaging agents suitable for a variety of cancer indications (*13*,*14*).

FAP-2286 comprises a peptide that potently and selectively binds to FAP and DOTA attached via a linker (*15*). FAP-2286 and its metal complexes had potent affinity to human FAP protein (dissociation constant, 0.4–1.4 nM), whereas limited off-target activity was seen against closely related family members. Labeled with the therapeutic β-particle–emitting radionuclide ^177^Lu, FAP-2286 had potent antitumor activity in FAP-expressing HEK-293 xenografts after a single intravenous dose (*16*).

Here, we report our initial experience with FAP-2286 radiolabeled with the β-particle emitter ^177^Lu for the treatment of patients with advanced metastatic cancers after exhaustion of all other treatment options.

## MATERIALS AND METHODS

### Patients and Regulatory Issues

Peptide-targeted radionuclide therapy (PTRT) with ^177^Lu-FAP-2286 was administered with palliative intent, after pretherapeutic confirmation of FAP expression of the metastases using ^68^Ga-FAP-2286 (*n* = 9) or ^68^Ga-FAPI-04 (*n* = 2) PET/CT (tumor-to-background [liver] SUV ratios > 3, Supplemental Table 1; supplemental materials are available at http://jnm.snmjournals.org), to 11 patients with progressive and metastatic adenocarcinoma of the pancreas (patients 1–5), breast (patients 6–9), ovary (patient 10), or rectum (patient 11). The administration was in accordance with paragraph 37 of the updated Declaration of Helsinki, “Unproven Interventions in Clinical Practice,” and with the German Medical Products Act (Arzneimittelgesetz §13 2b). Patients 5 and 9 were screened using ^68^Ga-FAPI-04 PET/CT before therapy, whereas all the other patients were screened by ^68^Ga-FAP-2286 PET/CT. The cutoff of at least 3 for tumor-to-liver SUV ratio on screening ^68^Ga-FAP-2286 PET/CT was to ensure a significant FAP expression of metastases as an essential criterion for patient selection, also considering the low physiologic liver uptake. PTRT was administered at Zentralklinik Bad Berka. The study was performed in accordance with German regulations (Federal Agency for Radiation Protection) concerning radiation safety and was approved by the institutional review board. All patients signed a detailed informed-consent form before undergoing the treatment and consented to the use of their anonymized clinical data for scientific purposes.

The patients were followed up until death or progression after initiation of PTRT. Metastases were present in the lymph node (*n* = 6), lung (*n* = 3), pleura (*n* = 1), peritoneum (*n* = 3), liver (*n* = 7), bone (*n* = 5), or diffusely in bone marrow (*n* = 2). Six patients had undergone surgery for their primary tumor, 8 had been treated with chemotherapy, 3 had received other radionuclide therapies, 2 had been treated with checkpoint inhibitors, and 1 had received a poly(adenosine diphosphate-ribose)polymerase inhibitor. Chemotherapy had been concluded at 4 wk before the start of PTRT in patient 9 and at 2 wk before PTRT in patients 2 and 7. Patient 10 received the last nivolumab administration 4 wk beforehand. Two patients (patients 1 and 3) categorically refused any other treatment, such as surgery or chemotherapy, and patient 5 was deemed unfit for chemotherapy. The patients’ characteristics are listed in Table 1.

**TABLE 1:** Patient Characteristics

Patient no.	Age (y)	Sex	Primary tumor	Hormone receptor status, if applicable	Metastases	Relevant previous surgery	Previous chemotherapy regimen	Previous radionuclide therapy, if applicable, with cumulative administered radioactivity	Other relevant treatments
1	70	M	Pancreas (head and body)		LN, hep, per, oss	None	None		None
2	55	F	Pancreas (tail)		LN	Left pancreatectomy	FOLFIRINOX, nab-paclitaxel, gemcitabine		None
3	78	M	Pancreas (head and tail)		Hep, per, oss	None	None		None
4	58	M	Pancreas (body)		LN, hep, oss	None	Abraxane, gemcitabine, capecitabine, oxaliplatin, irinotecan		None
5	87	M	Pancreas (body and tail)		LN, hep, per	None	None—unfit for chemotherapy		None
6	63	F	Breast	ER- and PR-positive; HER2-negative	Oss, LN, hep	Mastectomy	None	^177^Lu-labeled HER2-ligand and bisphosphonate; 4.5 GBq (122 mCi)	Hormonal therapy, EBRT to bone metastases
7	65	F	Breast	ER- and PR-positive; HER2-negative	Oss, hep, brain	Mastectomy	5-FU, epirubicin, und cyclophosphamide	^177^Lu-labeled bisphosphonate; 34.4 GBq (930 mCi)	Hormonal therapy, EBRT to bone and brain metastases
8	40	F	Breast	ER-, PR-, and HER2-positive	Oss, LN, hep, pul	Liver segment resection	Docetaxel, cyclophosphamide	^177^Lu-labeled HER2-ligand and bisphosphonate; 15.7 GBq (424 mCi)	Trastuzumab, EBRT to bone metastases
9	58	F	Breast		Oss, hep	Mastectomy	Docetaxel, doxorubicin, cyclophosphamide, capecitabine, 5-FU, methotrexate		EBRT to primary, hormonal therapy, palbociclib, chemoembolization of liver metastases
10	50	F	Ovary		Pleuroperitoneal, local bowel infiltration	Palliative bowel surgery	Cisplatin, paclitaxel, carboplatin		Bevacizumab, olaparib, nivolumab
11	61	M	Rectum		Hep, pul, LN	Resection of rectum, liver segment, and lung lobe	FOLFOX, 5-FU, irinotecan		Preoperative EBRT to primary, panitumumab, ramucirumab, pembrolizumab

LN = lymph node; hep = hepatic; per = peritoneal; oss = osseous; FOLFIRINOX = leucovorin, fluorouracil, irinotecan, and oxaliplatin; ER = estrogen receptor; PR = progesterone receptor; HER2 = human epidermal growth factor receptor 2; EBRT = external-beam radiation therapy; 5-FU = 5-fluorouracil; pul = pulmonary; FOLFOX = folinic acid, fluorouracil, and oxaliplatin.

### Radiolabeling of FAP-2286 with ^68^Ga

The peptide FAP-2286 was labeled with ^68^Ga using the NaCl-based labeling procedure as previously described (*17*). For the automated radiopharmaceutical production, a Modular-Lab PharmTracer Modul (Eckert and Ziegler) was used (*18*). In detail, up to 4 ^68^Ge/^68^Ga generators were eluted, and the eluate (1.2–2.6 GBq) was passed through a preconditioned strong-cation-exchange cartridge. The ^68^Ga collected was subsequently eluted using 0.5 mL of 5 M NaCl spiked with 12.5 μL of 5.5 M HCl. The ^68^Ga-eluate of the strong-cation-exchange cartridge was then added to a solution of 150 μg of FAP-2286, 5 mg of l-ascorbic acid, and 1.2 mg of l-methionine dissolved in 350 μL of 1 M sodium acetate buffer (adjusted with HCl and acetic acid to pH 4.5) and 2.3 mL of water for injection. The reaction mixture was heated to 95°C for 8 min. After labeling, the mixture was diluted with 2 mL of water for injection, neutralized using 2 mL of sterile sodium phosphate buffer (B. Braun Melsungen), and sterile-filtered. Samples were obtained for quality control, endotoxin testing, and sterility testing. The radiolabeling yield and the radiochemical purity were determined by instant thin-layer chromatography and high-performance liquid chromatography, respectively.

### Radiolabeling of FAP-2286 with ^177^Lu

Automated labeling was performed using a PiRoSyn synthesis module (Sykam) (*19*). To a solution of ^177^Lu in 300 μL of 0.05 M HCl, a solution of 10 mg of l-ascorbic acid, 5 mg of l-methionine, and FAP-2286 (20 MBq of ^177^Lu/μg of FAP-2286) in 1 mL of 1 M sodium acetate buffer (adjusted with HCl to pH 5.5) was added. The mixture was heated to 95°C for 35 min. The mixture was diluted with a 0.9% saline solution and sterile-filtered. Samples were tested for quality, endotoxins, and sterility. Radiochemical purity, as determined by thin-layer and high-performance liquid chromatography, was consistently higher than 98%.

### Treatment Protocol

^177^Lu-FAP-2286 was administered intravenously over 5–10 min. All patients presented with aggressive disease and a high tumor load, primarily necessitating high dosages (radioactivities to be administered) for tumor control. The injected activity was adapted, if necessary, on the basis of not only the patient’s clinical condition, hematologic results, and renal function but also the tumor distribution, that is, in cases of red marrow involvement. For example, a preexisting grade 2 anemia necessitated reduction of the radioactivity to be administered. When the patient consented and the patient’s condition allowed for further treatment, additional cycles were administered 8 wk later. One patient received a single cycle, 9 patients received 2 cycles, and 1 patient received 3 cycles (Supplemental Table 2).

For prevention of nausea, 3 mg of granisetron were injected intravenously before ^177^Lu-FAP-2286 administration. For adequate hydration, 1 L of a balanced electrolyte solution was administered for 2 h after radiopharmaceutical application, with the addition of 20 mg of furosemide. Symptoms and vital parameters were monitored before, during, and after treatment. Patient characteristics, tumor features, and all previous treatments were documented. Complete blood counts, liver and kidney function results, creatine kinase levels, uric acid levels, and electrolyte levels, as well as levels of tumor-associated markers such as carcinoembryonic antigen, carcinoma antigen 15-3, carbohydrate antigen 19-9, and carbohydrate antigen 125, were determined before and during follow-up after each PTRT. Hematologic toxicity was graded according to the Common Terminology Criteria for Adverse Events, version 5.0 (*20*).

### Scintigraphy and SPECT/CT Imaging

The kinetics of ^177^Lu-FAP-2286 were determined on the basis of planar whole-body scintigraphy studies (anterior/posterior) and SPECT/CT 18–46 h after administration of the radiopharmaceutical. Planar scintigraphy was acquired using a Spirit DH-V dual-head γ-camera (Mediso); medium-energy, general-purpose collimator; 15% energy window; 208-keV peak; and 15 cm/min speed. SPECT/CT was performed using a Symbia T camera system (Siemens Healthcare GmbH) with a medium-energy, low-penetration collimator; peaks at 113 keV and 208 keV (15% energy windows and 20% upper and lower scatter window); 128 × 128 matrix; 32 projections with 30 s per step; and body contour.

### Dosimetry Protocol

At least 5 serial planar whole-body scintigraphy studies and 1 regional SPECT/CT study were acquired per patient starting from 0.5 h (immediately after therapeutic administration and before bladder voiding) after injection. Since the patients were not allowed to empty the bladder before the first scan, the total-body counts acquired immediately after the injected activity were defined to be 100% of the administered activity. Total-body counts on the subsequent scans were expressed as fractions of injected activity. Regions of interest were drawn manually over the source regions on the acquired scintigraphy images, which were then analyzed using the software of the Hermes system (Hybrid Viewer). Source regions were defined as organs and metastases showing significant specific uptake, which could be clearly delineated on each posttherapy scan. Regions of interest were selected by the same physicist, in collaboration with a nuclear medicine physician, who selected the suitable lesions for dosimetry (i.e., lesions with the highest uptake in the respective organ). As representative tumor lesions for dosimetry, bone and liver metastases were chosen because of their accurate localization and measurability on whole-body and SPECT/CT scans, as well as the high frequency of bone metastases. Lymph node and peritoneal lesions are limited by overlap on whole-body scans. The kinetics of the whole body and the source organs were determined on the basis of this region-of-interest analysis. The SPECT/CT scans were reconstructed and quantified using the Hermes SUV SPECT software. After segmentation, the SPECT activity of source regions was used to scale the time–activity curves obtained from planar imaging. In the next step, these time–activity curves were fitted to mono- or biexponential functions to calculate effective half-lives and the time-integrated activity coefficient (Origin Pro 8.1G; OriginLab Corp.). Mean absorbed organ and tumor doses were finally estimated using OLINDA 2.0. The International Commission on Radiological Protection 89 adult model and the spheres model were used for normal organs and tumor lesions, respectively (both included in OLINDA 2.0). The model was adapted to individual normal-organ and tumor volumes obtained from the latest CT scan of the patient.

Dosimetry was estimated in 10 treatment cycles: a single cycle in patients 1, 4, 5, and 10 and 2 cycles in patients 6, 7, and 8. Posttherapy scans were acquired at defined mandatory time points: immediately after injection; 2–3 h after injection; and 1, 2, and 3 d after injection, as well as (when the patient’s condition so allowed) a further delayed scan up to 10 d after injection. Analysis was performed in accordance with our previously described protocol (*21*). Time-dependent activity in kidneys and metastases (13 osseous and 1 hepatic metastases) was determined by drawing regions of interest on serial ^177^Lu-FAP-2286 whole-body scans after therapy. The mean absorbed dose to the red marrow was estimated from activity in the blood in 4 of 10 estimations, when the general condition and vein characteristics of the patient allowed drawing of the required multiple blood samples. In the other 6 of 10 estimations, the mean absorbed dose to the red marrow was determined from the whole-body activity distribution.

### Clinical, Radiologic, and Laboratory Follow-up

Patient records were reviewed for any incidence of hematologic, gastrointestinal, renal, hepatic, or other adverse events; grade was assigned according to Common Terminology Criteria for Adverse Events, version 5.0. Circumstances that resulted in cessation of, or a delay in, treatment were documented. Changes in circulating tumor markers were also recorded. All patients were systematically followed up after therapy by determining relevant laboratory parameters every 2 wk. Baseline ^68^Ga-FAP-2286 PET/CT and contrast-enhanced CT or MRI were performed at a mean of 2 d (range, 1–7 d) before the first PTRT cycle. To determine treatment efficacy, at 6–8 wk after first therapy cycle CT/MRI was performed in all patients and ^68^Ga-FAP-2286 PET/CT was performed in 10 patients (the exception was patient 5). Patient 8 underwent restaging using ^68^Ga-FAP-2286 PET/CT at 8 wk after the third therapy cycle.

## RESULTS

### Tolerability

The mean ± SD of the administered amount of FAP-2286 was 290 ± 100 μg (range, 120–495 μg). The mean administered activity was 5.8 ± 2.0 GBq (range, 2.4–9.9 GBq). There were no adverse or clinically detectable pharmacologic effects in any of the 11 patients. No significant changes in vital signs or in the results of laboratory studies, concerning immediate adverse events, were observed. Patients 4 and 6 reported a significant improvement in pain after treatment, requiring less morphine; patient 5 had an improvement in physical capacity and self-reported quality of life after treatment, in addition to pain relief.

### Posttherapeutic ^177^Lu-FAP-2286 Whole-Body Scans and SPECT/CT

**FIGURE 1.**
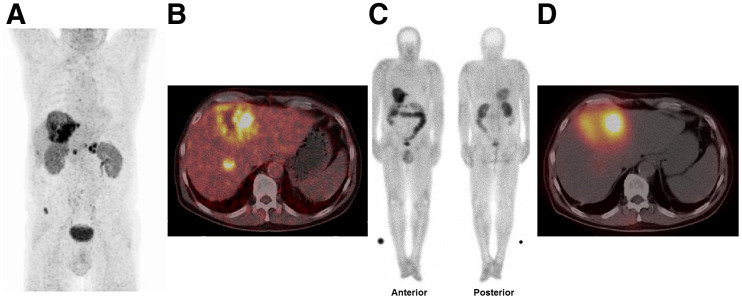
(A and B) Patient 4 had adenocarcinoma of pancreatic body, as well as hepatic, peripancreatic lymph node, and osseous metastases, which demonstrated high FAP expression on maximum-intensity-projection ^68^Ga-FAP-2286 PET image (A) and transverse ^68^Ga-FAP-2286 PET/CT image (B). (C and D) Significant uptake and late retention of ^177^Lu-FAP-2286 were noted in liver metastases on posttherapeutic whole-body scintigraphy in anterior and posterior views at 48 h after injection (C) and on transverse SPECT/CT image (D). Because of low resolution of ^177^Lu for imaging, as compared with ^68^Ga for PET/CT, not all tumor sites seen on ^68^Ga-FAP-2286 PET/CT are apparent on ^177^Lu-FAP-2286 images.

**FIGURE 2.**
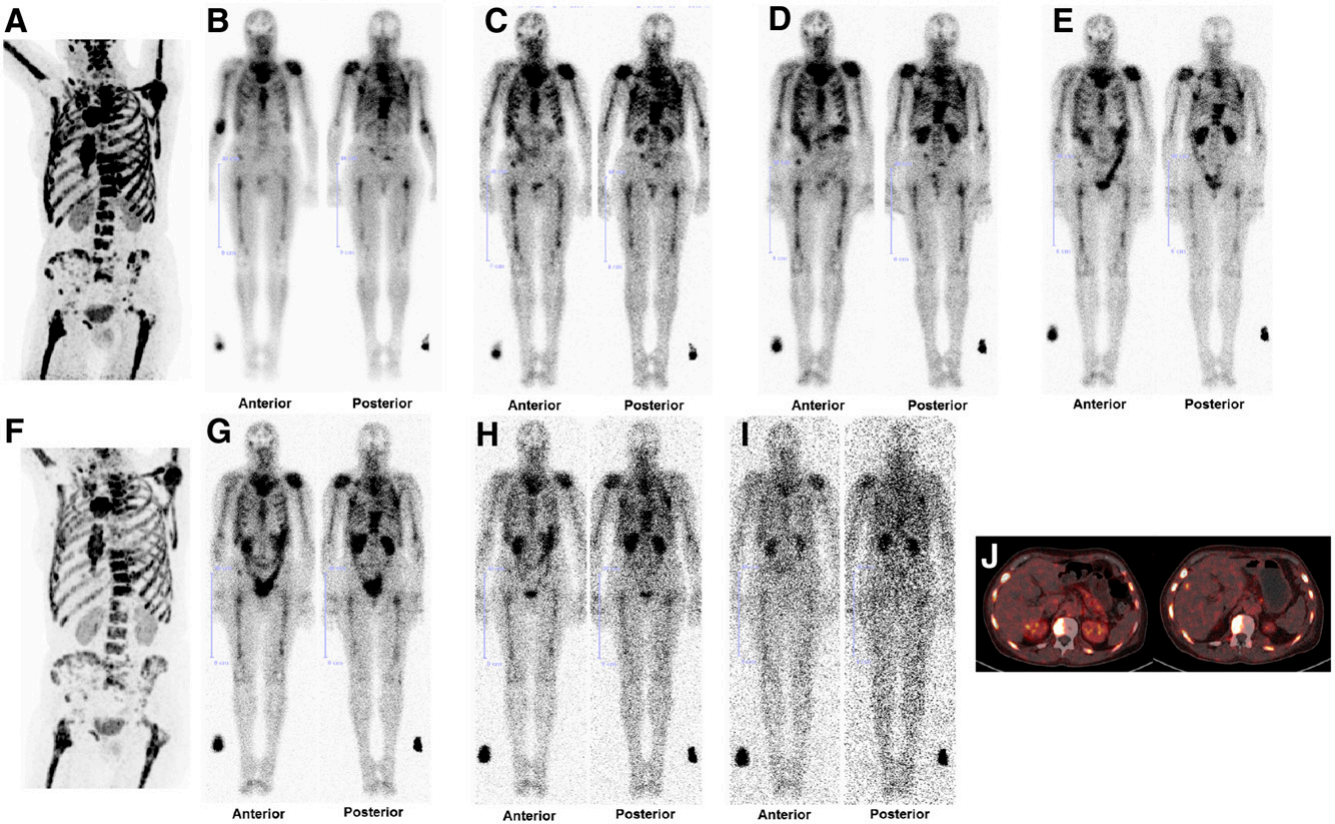
(A) Patient 6, with breast cancer, presented predominantly with diffuse FAP-positive bone and bone marrow metastases (but also lymph node metastases) on ^68^Ga-FAP-2286 PET/CT. (B, C, D, E, G, H, and I) Serial whole-body scintigraphy in anterior and posterior views at 3 h (B), 20 h (C), 44 h (D), 68 h (E), 92 h (G), 7 d (H), and 10 d (I) after PTRT using 2.4 GBq of ^177^Lu-FAP-2286 demonstrated uptake and retention in metastases. (F) ^68^Ga-FAP-2286 PET/CT after 8 wk demonstrated mixed response: regression of bone and bone marrow lesions but overall progressive disease with new evidence of liver metastases. (J) Axial ^68^Ga-FAP-2286 PET/CT images before (left) and after (right) PTRT show FAP-positive metastases in ribs and vertebrae, and occurrence of a new liver metastasis on the right side.

Visual analysis of posttherapy whole-body scans and SPECT/CT scans demonstrated significant uptake and retention of ^177^Lu-FAP-2286 in tumor lesions on delayed imaging (72 h–10 d after injection) in all patients (SUVs of ^177^Lu-FAP-2286 in tumor lesions are displayed in Supplemental Table 3, and those of the kidneys are in Supplemental Table 4; representative examples are shown in Figs. 1 and 2, and the whole-body scans of patients 1–3, 5, and 7–11 are in Supplemental Fig. 1). The initial biodistribution of the radiopharmaceutical was identical to that of the pretherapeutic ^68^Ga-FAP-2286 PET/CT.

### Dosimetry

Dosimetric parameters, specifically effective half-life and mean absorbed dose to whole body, kidneys, and red marrow (Supplemental Table 5), as well as metastases (Supplemental Table 6), expressed as mean ± SD, were determined after ^177^Lu-FAP-2286 administration (Supplemental Table 7 lists the dose estimations for all organs). The effective half-lives were as follows: whole body, 35 ± 9 h (range, 25–48 h); kidneys, 81 ± 51 h (range, 30–161 h); and bone metastases (*n* = 13), 44 ± 25 h (range, 21–120 h). The mean absorbed doses were as follows: whole body, 0.07 ± 0.02 Gy/GBq (range, 0.04–0.1 Gy/GBq); kidneys, 1.0 ± 0.6 Gy/GBq (range, 0.4–2.0 Gy/GBq); red marrow, 0.05 ± 0.02 Gy/GBq (range, 0.03–0.09 Gy/GBq); and bone metastases, 3.0 ± 2.7 Gy/GBq (range, 0.5–10.6 Gy/GBq). The effective half-life and mean absorbed dose in 1 liver metastasis (in patient 4) were estimated to be 32 h and 0.4 Gy/GBq, respectively.

### Adverse Events

Clinical adverse events included grade 1 (G1) short-term and self-limiting headache in patients 3, 7, 9, and 10 within 12 h after treatment, and moderate headache (grade 2 [G2]) in patient 9. There was a severe flare-up of abdominal pain (grade 3 [G3]), with nausea and vomiting, after the second cycle in patient 2. Incidentally, this patient had reported a significant reduction in breathing-related pain in the left side of the back after the first cycle.

New anemia occurred after PTRT in 3 patients (G1 in patients 1 and 4; G2 in patient 11). Worsening of a preexisting G1 anemia to G2 was noted in patients 5 and 7 after the second cycle. After the third cycle, patient 8 experienced G3 pancytopenia (from preexisting G1–G2), requiring packed red cell transfusions and granulocyte–colony-stimulating factor. Preexisting leukocytopenia worsened from G1 to G2 in patient 5 and from G2 to G3 in patient 6. After PTRT, there was leukocytosis (non-G3) in patients 3, 4, 7, and 11; in patients 3, 7 and 11, it occurred after the first cycle and was reversible. In patient 4, however, it occurred after the second cycle and persisted until death 8 wk later. Interestingly, in patient 9, the leukocyte counts normalized (from preexisting G1 leukocytopenia) and the absolute thrombocyte count improved (G1 unchanged) at 6 wk after the first PTRT cycle (Table 2; Supplemental Table 8).

**TABLE 2:** Safety: Hematologic and Renal Function Before and 6–8 Weeks After ^177^Lu-FAP-2286 PTRT According to Common Terminology Criteria for Adverse Events, Version 5.0

	Hemoglobin (mmol/L)			Leukocyte count (billion cells/L)			Thrombocyte count (billion cells/L)			eGFR (mL/min/1.73 m^2^)		
Grade	Before therapy (*n* = 11)	After first PTRT (*n* = 11)	After second PTRT (*n* = 8)	Before therapy (*n* = 11)	After first PTRT (*n* = 11)	After second PTRT (*n* = 8)	Before therapy (*n* = 11)	After first PTRT (*n* = 11)	After second PTRT (*n* = 8)	Before therapy (*n* = 11)	After first PTRT (*n* = 11)	After second PTRT (*n* = 8)
1	4	5	3	2	1	0	5	3	5	0	0	0
2	2	3	4	2	2	2	0	0	0	1	1*	1*
3	0	0	0	0	0	1	0	0	0	0	0	0
4	0	0	0	0	0	0	0	0	0	0	0	0
5	NA	0	0	NA	0	0	NA	0	0	NA	0	0

*There was additional non-G3 acute (on preexisting chronic G2) prerenal renal insufficiency after both first and second cycles, which was reversible.

eGFR = estimated glomerular filtration rate; NA = not applicable before ^177^Lu-FAP-2286 PTRT (grade 5 represents death).

With preexisting chronic renal insufficiency (G2), patient 6 had episodes of acute deterioration of renal function after the first and second PTRT cycles. Both episodes were determined to be prerenal in origin, i.e., a sudden reduction in blood flow to the kidney (renal hypoperfusion) caused a loss of kidney function. In prerenal kidney injury, there is nothing wrong with the kidney itself, and in both instances, renal function returned to normal within 4 wk and the glomerular filtration rate remained consistently above 30 mL/min. There was no significant change in renal parameters in the rest of the patients. The hepatic function parameters, uric acid levels, electrolyte levels, and creatinine kinase levels were unaffected by PTRT.

### Outcome After PTRT

Evaluation according to RECIST 1.1 in all patients at 6–8 wk after the first PTRT cycle revealed stable disease in patients 8 and 9 and progression in the other 9 patients (Table 3). The findings also correlated with circulating tumor markers (Supplemental Table 9) and with ^68^Ga-FAP-2286 PET/CT findings (performed on 10 patients). Because of marked disease progression, no further PTRT was administered to patient 11. In patient 6, ^68^Ga-FAP-2286 PET/CT revealed a mixed response (i.e., remission of the diffuse bone metastases), but overall disease was progressive, with evidence of new hepatic lesions (Fig. 2). Patient 8 demonstrated progression at 8 wk after the third PTRT cycle. Six patients died from disease progression 2–8 mo after their initial PTRT (Table 3). One patient died from suicide and 1 from pneumonia.

**TABLE 3:** Outcome After Treatment

Patient no.	Initial diagnosis	First PTRT cycle	FAP PTRT cycles (*n*)	Response* at 6–8 wk after first cycle	Response* at 6–8 wk after third cycle, if applicable	Time to progression since initial PTRT (wk)	Death	Cause of death	Survival since initial FAP PTRT (mo)
1	09/2019	10/2019	2	PD		8	03/2020	PD	5
2	03/2019	10/2019	2	PD		8	06/2020	PD	8
3	09/2019	10/2019	2	PD		8	12/2019	Suicide	2
4	02/2018	10/2019	2	PD		8	02/2020	PD	4
5	01/2019	12/2019	2	PD^†^		8	04/2020	PD	4
6	07/2015	10/2019	2	PD		8			Patient alive (05/2021)
7	09/2004	10/2019	2	PD		8	04/2020	PD	6
8	07/2013	10/2019	3	SD	PD	24			Patient alive (05/2021)
9	05/2008	10/2019	2	SD		20			Patient alive (05/2021)
10	03/2015	10/2019	2	PD		8	01/2020	PD	3
11	02/2009	10/2019	1	PD		6	01/2020	Bronchopneumonia	3

*Response was evaluated by RECIST 1.1, on ^68^Ga-FAP-2286 PET/CT (except in patient 5) and tumor marker evaluation after first (in all) and third (in patient 8) PTRT cycles.

^†^Response was evaluated on RECIST 1.1 and tumor marker evaluation.

FAP = fibroblast activation protein; PTRT = peptide-targeted radionuclide therapy; PD = progressive disease.

## DISCUSSION

This retrospective report provides the first—to our knowledge—evidence of the feasibility of theranostics in diverse advanced adenocarcinomas using the novel radiolabeled peptide ^177^Lu-FAP-2286. PTRT was performed on a compassionate-use basis in end-stage pancreatic, breast, ovarian, and colorectal cancer patients after exhaustion of the treatment options, including 2 patients who had strictly refused any other treatment and 1 patient who was deemed unfit for chemotherapy. The first-in-humans use demonstrated a favorable safety profile with few manageable serious adverse events.

Pancreatic, breast, ovarian, and colorectal adenocarcinomas and their metastases have been demonstrated to be FAP-positive on PET/CT (*22*). In our patient cohort, FAP expression was confirmed in these malignancies on PET/CT using ^68^Ga-FAP-2286 or ^68^Ga-FAPI-04. Biodistribution images after therapy revealed not only significant tumor uptake of ^177^Lu-FAP-2286 but also long retention of the radiopharmaceutical in all patients. In contrast, biodistribution studies with small-molecule–based FAPI tracers, notably FAPI-02 and FAPI-04, revealed an earlier tumor washout and a correspondingly shorter tumor retention time (*14*), thereby limiting their therapeutic potential when conjugated to longer-lived therapeutic radionuclides such as ^177^Lu. Thus, the application of shorter-lived radionuclides for therapy using FAPI-based tracers was suggested (*19*). The longer tumor retention of FAP-2286, on the other hand, allows the use of therapeutically effective longer-lived radionuclides for therapy (including ^177^Lu and ^225^Ac).

These findings were further corroborated by dosimetric studies. Comparison of ^177^Lu-FAP-2286 to other radiopharmaceuticals previously reported to be effective (i.e., ^177^Lu-DOTATATE for neuroendocrine tumors (*23*) and ^177^Lu-PSMA-617 for prostate cancer (*24*)) shows comparable absorbed doses for whole body, bone marrow, and kidneys (*21*,*25*,*26*). Notably, the kidney absorbed dose delivered by ^177^Lu-FAP-2286 when used without renal protection was comparable to that of ^177^Lu-PSMA-617 and to that delivered by ^177^Lu-DOTATATE when coadministered with Lys/Arg for renal protection (1.0 Gy/GBq [range, 0.4–2.0 Gy/GBq] vs. 0.99 Gy/GBq [range, 0.45–1.6 Gy/GBq] vs. 0.8 Gy/GBq [range, 0.3–2.6 Gy/GBq]) (*21*,*27*). Whole-body and bone marrow absorbed doses of ^177^Lu-FAP-2286 (0.07 and 0.05 Gy/GBq, respectively) were similar to those of ^177^Lu-PSMA-617 (0.04 and 0.03 Gy/GBq, respectively) (*25*) and ^177^Lu-DOTATATE (0.05 and 0.04 Gy/GBq, respectively) (*21*,*26*). Absorbed doses in bone metastases were similar to those reported by Kulkarni et al. for ^177^Lu-PSMA-617 (3.0 Gy/GBq [range, 0.5–10.6 Gy/GBq] for ^177^Lu-FAP-2286 vs. 2.9 Gy/GBq for ^177^Lu-PSMA-617) (*25*) and slightly lower than those reported by Violet et al. for ^177^Lu-PSMA-617, although with an overlapping range (5.3 Gy/GBq [range, 0.4–10.7 Gy/GBq]) (*28*). The effective half-life of ^177^Lu-FAP-2286 in the whole body (35 h) was slightly shorter than that of ^177^Lu-PSMA-617 (40 h) (*25*) and ^177^Lu-DOTATATE (55 h) (*21*) and slightly longer in the kidneys (81 h for ^177^Lu-FAP-2286 vs. 42 h for ^177^Lu-PSMA-617 and 63 h for ^177^Lu-DOTATATE) (*21*,*25*). Thus, although individual values may vary, the overall dose delivered by ^177^Lu-FAP-2286 to healthy organs and tumor lesions appears comparable to that of well-known ^177^Lu-based radiopharmaceuticals. The dosimetric analysis of the patients in this study therefore justifies prospective clinical trials to establish a safe and effective cumulative dose of ^177^Lu-FAP-2286, as well as risk factors to be considered in diverse adenocarcinoma patients.

PTRT with ^177^Lu-FAP-2286 appears to ameliorate symptoms in rapidly proliferating adenocarcinomas as noted by significant pain reduction in patient 4, with liver metastases; patient 6, with diffuse bone metastases; and patient 5, with newly diagnosed pancreatic cancer. On the other hand, there was exacerbation of preexisting pain or development of new local symptoms shortly after PTRT in patient 9 (headache) and in patient 2 after the second cycle (severe abdominal complaints). A flare phenomenon after treatment with radionuclide therapy has been reported previously (*29*); thus, the symptoms in patients 2 and 9 may potentially be related to existing metastases localized in the area of pain exacerbation.

The reasons for a reduction in the radioactivity administered were preexisting red marrow dysfunction (anemia or pancytopenia)—either because of red marrow involvement or because the patient had previously undergone multiple previous therapies, including chemotherapy—and renal dysfunction, which potentially could result in a longer whole-body residence time and increase the absorbed dose to red marrow. Therefore, the presence of these limitations prevented use of a higher injected activity to prevent potential additional red marrow dysfunction. G3 hematologic adverse events were observed in patient 6 (leukocytopenia) and patient 8 (pancytopenia), correlating with imaging evidence of diffuse bone marrow involvement. These adverse events were managed with intermittent transfusions of packed red blood cells and bone marrow stimulation using granulocyte–colony-stimulating factor. Otherwise, no severe hematologic toxicity was noted in patients, despite heavy pretreatment in some, including recently concluded chemotherapy (patients 2, 7, and 9), immune-checkpoint-inhibitor therapy (patient 10), and radionuclide therapies targeting human epidermal growth factor receptor 2 or bone (patients 6, 7, and 8). In fact, the bone marrow function in patient 9 not only was unaffected by PTRT but improved (most probably because of termination of chemotherapy 2 wk before the start of PTRT). No organ toxicity was seen. The one instance of prerenal, acute-on-chronic renal insufficiency in patient 6 could be managed with intravenous fluids and was resolved after 1 wk.

The most likely mechanism of action of PTRT can be postulated to be the destruction of cancer-associated fibroblasts, which are the support system for cancers, as well as a crossfire effect causing tumor-cell killing. However, progression of disease was frequently observed, necessitating alteration of the treatment strategy. Possible measures to enhance the therapeutic efficacy of PTRT are a shortening of the intervals between treatments or an increase in the administered radioactivity. Furthermore, the evaluation of combinations with other treatments (e.g., with a targeted therapy such as poly(adenosine diphosphate-ribose)polymerase inhibitor or immune checkpoint inhibitor) stands to reason.

Other radionuclides for labeling FAP-2286 may be used for therapy. Although the β-particle energy of ^90^Y is higher than that of ^177^Lu, the longer range of ^90^Y-β might increase the risk of bone marrow and renal toxicity. However, applying a lower dosage could be appropriate, as with PRRT of neuroendocrine tumors (*27*). An α-emitter, such as ^225^Ac, appears to also be a suitable candidate because of its high and precise energy delivery to the tumor per unit of radioactivity, causing double-stranded DNA breaks (*30*). A proof-of-concept study of ^225^Ac-FAPI-04 (as well as ^64^Cu-FAPI-04) in FAP-expressing pancreatic cancer xenograft mouse models suggested the applicability of FAP-targeted α-therapy in pancreatic cancer (*31*). The tumor half-life of FAP-2286 is much longer than that of FAPI-02 or FAPI-04 but less than that of ^177^Lu-PSMA, ^177^Lu-DOTATOC, or ^177^Lu-DOTATATE (*14*,*21*,*25*). In consequence, given the effective tumor half-life of ^177^Lu-FAP-2286 (mean, 44 h for bone and 32 h for single liver metastases), radionuclides such as ^67^Cu or ^90^Y (which has the additional advantage of a higher β-particle energy) may increase exposure as compared with ^177^Lu or ^225^Ac. Further studies using these radionuclides would be beneficial.

The major limitation of this study was the small and heterogeneous patient population, which received as the last line of treatment PTRT with ^177^Lu-FAP-2286 on a compassionate-use basis. This was not a dose escalation study, and varying radioactivities of ^177^Lu-FAP-2286 administered to patients make safety and therapeutic assessment only observational. Therefore, the preliminary but encouraging results of this retrospective analysis must be confirmed by a prospective, randomized, and controlled clinical trial.

## CONCLUSION

This study provides the first clinical evidence of the feasibility of treating different aggressive adenocarcinomas with ^177^Lu-labeled FAP-2286. High uptake and long retention in primary and metastatic tumor lesions and a reasonable toxicity profile warrant further investigation of ^177^Lu-FAP-2286 in prospective clinical studies to systematically evaluate its safety and efficacy and to define the patient population it would most benefit.

## DISCLOSURE

Christiane Smerling, Dirk Zboralski, Frank Osterkamp, Aileen Hoehne, and Ulrich Reineke are employees of 3B Pharmaceuticals and inventors on a patent application for FAP tracers. No other potential conflict of interest relevant to this article was reported.

## Notes

KEY POINTS **QUESTION:** Is the ^177^Lu-FAP-2286 therapy of different adenocarcinomas feasible in terms of retainability of tracer in the tumor, patient safety, and treatment efficacy? **PERTINENT FINDINGS:** In a series of 11 patients with diverse advanced adenocarcinomas, ^177^Lu-FAP-2286 was well tolerated, without any issues of urgent concern. This novel conjugate of a FAP-binding peptide demonstrated high uptake and long retention in primary and metastatic tumor lesions, with a reasonable toxicity profile. **IMPLICATIONS FOR PATIENT CARE:** This first-in-humans use provides a direction to clinical trials for promising and effective FAP-targeted radiopeptide therapy in various aggressive cancers.
